# Supplementary material for: Chromosomal instability can favor macrophage-mediated immune response and induce a broad, vaccination-like anti-tumor IgG response
Source: bioRxiv. 2023 Apr 4:2023.04.02.535275. Preprint. [Version 1] doi: 10.1101/2023.04.02.535275 (PMC10103980; doi:10.1101/2023.04.02.535275)

bioRxiv preprint doi: https://doi.org/10.1101/2023.04.02.535275; this version posted April 4, 2023. The copyright holder for this preprint (which was not certified by peer review) is the author/funder, who has granted bioRxiv a license to display the preprint in perpetuity. It is made available under aCC-BY 4.0 International license.

Supplementary Figure Legends

Supplementary Figure 1. Characterization of MPS1i-induced genome and chromosomal instability in B16F10 mouse melanoma.

(A) Quantification of micronuclei induced by different MPS1 inhibitors (AZ3146, BAY 12-17389, and reversine) at different concentrations. Statistical significance was calculated by ordinary one-way ANOVA and Tukey’s multiple comparison test (* $p < 0.05$; ** $p < 0.01$; *** $p < 0.001$; **** $p < 0.0001$).

(B) Consensus clustering of B16F10 analyzed in single-cell RNA-sequencing. B16F10 cells were treated with 2.5 µM MPS1i (reversine) or the equivalent volume of DMSO vehicle control. Cells were treated for 24 h, after which they were washed twice with PBS and allowed to recover for an additional 48 h. Cells were then collected and processes using 10X Genomics Chromium Single Cell Gene Expression kit for RNA isolation and library preparation.

(C) (i) UMAP plots of expression profiles for all B16F10 analyzed in single-cell RNA-sequencing. Top: UMAP plots depict clusters in which B16F10 fall, as determined by consensus clustering. Bottom: UMAP plots highlight cells with detectable copy number variations (CNVs), as determined by InferCNV. Red circles indicate individual cells with at least one CNV. Gray circles represent individual cells with no detectable CNV, suggesting these are chromosomally stable. **(ii)** Quantification of the composition (MPS1i-treated or DMSO-treated B16F10) of each cluster.

Supplementary Figure 2. Characterization of MPS1i-induced genome and chromosomal instability in B16F10 mouse melanoma.

(A) Flow cytometry analysis of CD47 and Tyrp1 expression on B16F10 mouse melanoma cells. Representative histograms for anti-CD47 (left) and anti-Tyrp1 (right) binding to B16F10 CD47 knockout (KO) and sgRNA ctrl (wild-type CD47 expression). Binding was detected by using secondary antibodies conjugated with Alexa Fluor 647.

(B) Phagocytosis of B16F10 cells in in a standard phagocytosis assay on two-dimensional tissue culture plastic, with or without the following: IgG opsonization by anti-Tyrp1, CD47 KO, and MPS1i-treatment of B16F10. B16F10 cells were treated with MPS1i or DMSO prior to this assay, following the same protocol described in Fig. 1A. **(i)** Schematic of the 2D phagocytosis assay. **(ii)** Representative images of phagocytosis. B16F10 cells (green) were incubated with

bioRxiv preprint doi: https://doi.org/10.1101/2023.04.02.535275; this version posted April 4, 2023. The copyright holder for this preprint (which was not certified by peer review) is the author/funder, who has granted bioRxiv a license to display the preprint in perpetuity. It is made available under aCC-BY 4.0 International license.

opsonizing anti-Tyrp1 or mouse IgG2a control and added to adherent bone marrow-derived macrophages (magenta). Random fields were imaged and then quantified to determine the percentage of phagocytic macrophages. Yellow arrowheads denote phagocytic events (complete engulfment of a target B16F10 cells). **(iii)** Quantification of phagocytic macrophages. Statistical significance was calculated by three-way ANOVA and Tukey's multiple comparison test. Mean ± SD shown, n = 3 replicates per condition (** $p < 0.01$; **** $p < 0.0001$).

(C) Quantification of cell size measurements by flow cytometry in both MPS1i- and DMSO-treated cells. Statistical significance was calculated by three-way ANOVA and Tukey's multiple comparison test. Mean ± SD shown, n = 3 replicates per condition (* $p < 0.05$; ** $p < 0.01$; *** $p < 0.001$).

(D) Quantification of Tyrp1 expression by flow cytometry in both MPS1i- and DMSO-treated cells. Statistical significance was calculated by three-way ANOVA and Tukey's multiple comparison test. Mean ± SD shown, n = 3 replicates per condition (**** $p < 0.0001$).

(E) Quantification of CD47 expression by flow cytometry in both MPS1i- and DMSO-treated B16F10 sgCtrl cells. Statistical significance was calculated by three-way ANOVA and Tukey's multiple comparison test. Mean ± SD shown, n = 3 replicates per condition (**** $p < 0.0001$).

Supplementary Figure 3. Macrophages readily clear CIN-afflicted tumoroids but only if CIN is accompanied by proliferation deficits.

(A) Timeline and schematic for generating engineered "immuno-tumoroids" for time-lapsed studies of macrophage-mediated phagocytosis of cancer cells. Tumoroids are formed by plating and culturing B16F10 cells on non-adhesive surfaces in U-bottom shaped wells. Prior to plating for tumoroid formation, B16F10 were treated with either MPS1i or DMSO (AZ3146, BAY 12-17389 or reversine). Tumoroid growth was measured by calculating the GFP+ area at the indicated timepoints (mean ± SD, n = 16 total tumoroids from two independent experiments for each condition). All data were then normalized to average GFP+ area on day 1 of each drug treatment's respective mouse IgG2a isotype control condition. Immuno-tumoroids are imaged at the listed timepoints. **(B)** To assess macrophage-mediated clearance, ~24 h after plating, bone marrow-derived macrophages (BMDMs) with or without opsonizing anti-Tyrp1 are added to the cohesive B16F10 tumoroids. Tumoroid growth curves for DMSO-treated B16F10 CD47 knockout (KO) cells. **(C)** Tumoroid growth curves (with BMDMs) for MPS1i-treated B16F10

bioRxiv preprint doi: https://doi.org/10.1101/2023.04.02.535275; this version posted April 4, 2023. The copyright holder for this preprint (which was not certified by peer review) is the author/funder, who has granted bioRxiv a license to display the preprint in perpetuity. It is made available under aCC-BY 4.0 International license.

CD47 KO cells (treated with varying concentrations of MPS1i): **(i)** AZ3146, **(ii)** BAY 12-17389. **(D)** Tumoroid growth curves (without BMDMs) for MPS1i-treated B16F10 CD47 KO cells (treated with varying concentrations of MPS1i): **(i)** BAY 12-17389, **(ii)** reversine. Both BAY 12-17389 and reversine induce proliferation deficits, which may play an essential role in mediating clearance by macrophages that could not be observed in AZ3146-treated cells.

Supplementary Figure 4. MPS1i-induced chromosomal instability upregulates MHC-1 class molecules on B16F10, suggesting increased antigen presentation.
Flow cytometry analysis and quantification H-2K^b expression on B16F10 mouse melanoma cells. B16F10 cells were treated with MPS1i or the equivalent volume of DMSO vehicle control. Concentrations used: 2.5 μM AZ3146, 1.0 μM BAY 12-17380, and 2.5 μM reversine. Cells were treated for 24 h, after which they were washed twice with PBS and allowed to recover for an additional 48 h. Binding was detected by using primary antibodies conjugated with Alexa Fluor 647. Statistical significance was calculated by Brown-Forsythe and Welch ANOVA and Dunnett's T3 multiple comparison test. Mean ± SEM shown, n = 3 independent replicates (* $p < 0.05$; ** $p < 0.01$; *** $p < 0.001$; **** $p < 0.0001$).

Supplementary Figure 5. Flow cytometry gating strategy for identification & quantification of macrophage infiltrate and characterization in CIN-afflicted and chromosomally stable B16F10 tumors.
Representative flow cytometry gating strategy for *in vivo* B16F10 CD47 KO tumor immune infiltrate five days after initial challenge. Tumors were comprised of cells treated with either **(i)** DMSO or **(ii)** MPS1i (2.5 μM), following the timeline schema in Fig. 2F. Singlets were separated from debris, doublets, and aggregates by FSC-A vs FSC-H and SSC-A vs SSC-H gates. Debris and dead cells were further removed by FSC-A vs SSC-A gating. Overall immune cell infiltrate was determined by CD45+ expression. Macrophages were then further identified based on Ly6G- and F4/80+ expression. Upon identification of macrophages, cells were further characterized for polarization based on CD86 (M1-like marker) and CD206 (M2-like marker) expression.

bioRxiv preprint doi: https://doi.org/10.1101/2023.04.02.535275; this version posted April 4, 2023. The copyright holder for this preprint (which was not certified by peer review) is the author/funder, who has granted bioRxiv a license to display the preprint in perpetuity. It is made available under aCC-BY 4.0 International license.

Supplementary Figure 6. MPS1i-induced CIN favors clearance when paired with CD47 KO and IgG opsonization, regardless of the degree of CIN.

Tumor growth curve of projected tumor area versus days after tumor challenge, with B16F10 CD47 KO cells. Each line represents a separate tumor and is fit with an exponential growth equation: $A = A_0e^{kt}$. Complete anti-tumor responses in which a tumor never grew are depicted with the same symbol as their growing counterparts and with solid lines at A = 0.

(A) Tumor growth curve data in which there was no complete response, from Fig. 3C. n = 7 mice that were challenged with DMSO-treated B16F10 CD47 KO and subsequently treated with mouse IgG2a control, n = 9 mice that were challenged with DMSO-treated B16F10 CD47 KO and subsequently treated with anti-Tyrp1.

(B) Tumor growth curve data from mice challenges with CIN-afflicted B16F10, but with varying concentration of MPS1i used to cause different degrees of instability. All mice here were challenged with B16F10 CD47 KO and treated with anti-Tyrp1 for conditions of maximal phagocytosis and to better compare to the ~97% cure rate in Fig. 3C, 4D-ii. n = 6 mice challenged with cells with each tested MPS1i concentration: 0.1, 0.5, 1.0 or 2.5 μM.

(C) Survival curves of mice up to 100 days after the tumor challenges in (A) and (B).

Supplementary Figure 7. Survivors challenged with CIN-afflicted tumors generate anti-cancer IgG, regardless of the degree of CIN.

Median fluorescence intensity quantification of IgG2a/c and IgG2b binding from sera from surviving mice with different degrees of chromosomal instability by titrating reversine concentration used to treat B16F10 CD47 KO cells before injection. Convalescent sera are collected from survivors from Fig. S6. Binding of IgG2a/c and IgG2b against **(A)** B16F10 cells expressing Tyrp1 and **(B)** B16F10 Tyrp1 knockout (KO). n = 4-5 distinct sera samples collected from survivors.

Supplementary Figure 8. Flow cytometry gating strategy for identification & quantification of immune infiltrate and characterization in re-challenge experiments.

Representative flow cytometry gating strategy for *in vivo* B16F10 CD47 KO tumor immune infiltrate from 2nd challenge non-survivors and age-matched naïve controls. Singlets were separated from debris, doublets, and aggregates by FSC-A vs FSC-H and SSC-A vs SSC-H

bioRxiv preprint doi: https://doi.org/10.1101/2023.04.02.535275; this version posted April 4, 2023. The copyright holder for this preprint (which was not certified by peer review) is the author/funder, who has granted bioRxiv a license to display the preprint in perpetuity. It is made available under aCC-BY 4.0 International license.

gates. Debris and dead cells were further removed by FSC-A vs SSC-A gating. For T cell quantification: CD45+ cells were gated on and then analyzed for CD3e and CD8a surface protein expression. For macrophage quantification: myeloid cells were isolated by CD45+ and CD11b+ expression, after which macrophages were isolated based on F4/80 expression.

bioRxiv preprint doi: https://doi.org/10.1101/2023.04.02.535275; this version posted April 4, 2023. The copyright holder for this preprint (which was not certified by peer review) is the author/funder, who has granted bioRxiv a license to display the preprint in perpetuity. It is made available under aCC-BY 4.0 International license.

Supplementary Figure 1

Induction of micronuclei and chromosomal instability in B16F10 by MPS1i

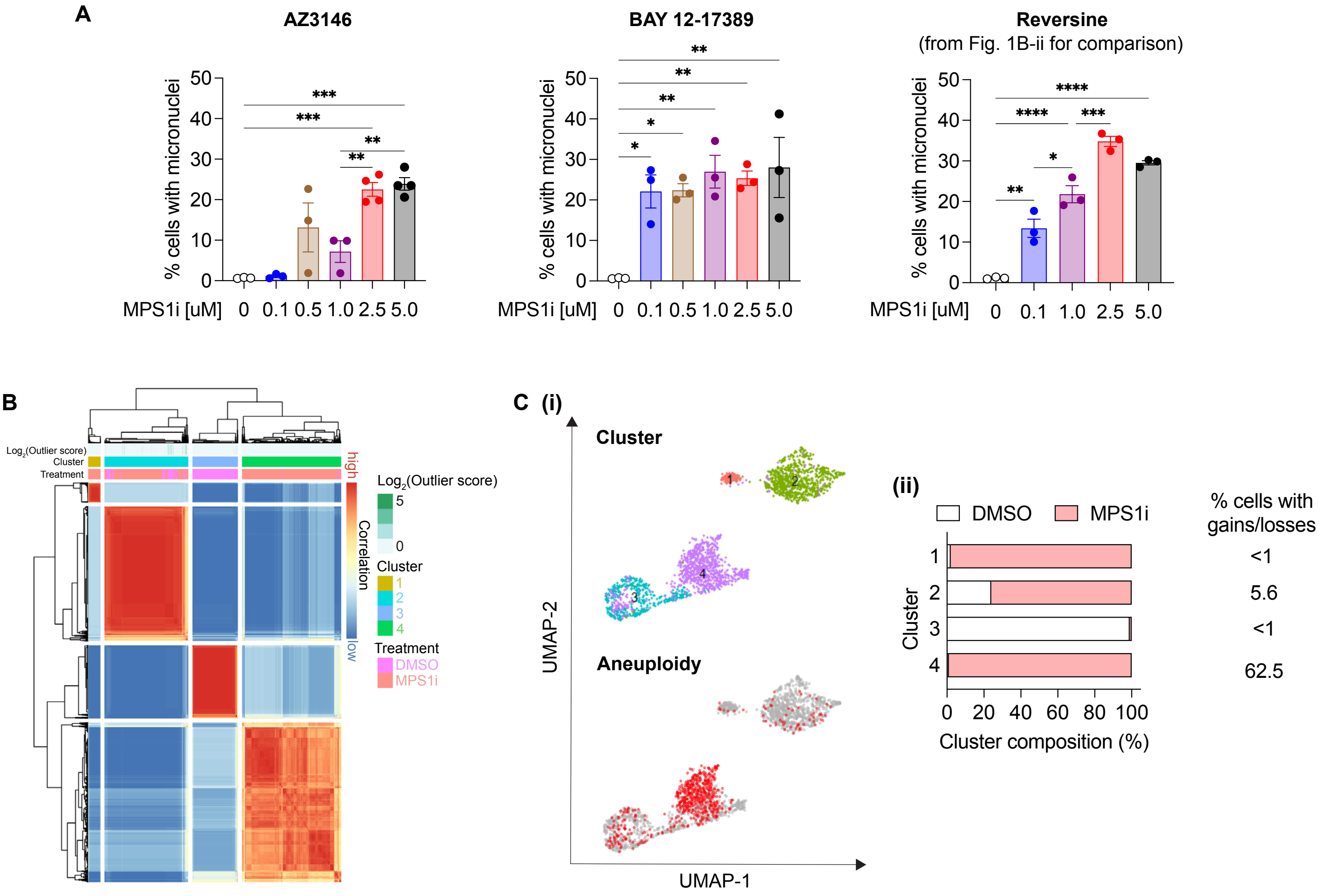

bioRxiv preprint doi: https://doi.org/10.1101/2023.04.02.535275; this version posted April 4, 2023. The copyright holder for this preprint (which was not certified by peer review) is the author/funder, who has granted bioRxiv a license to display the preprint in perpetuity. It is made available under aCC-BY 4.0 International license.

Supplementary Figure 2

Characterization & validation of factors affecting phagocytosis

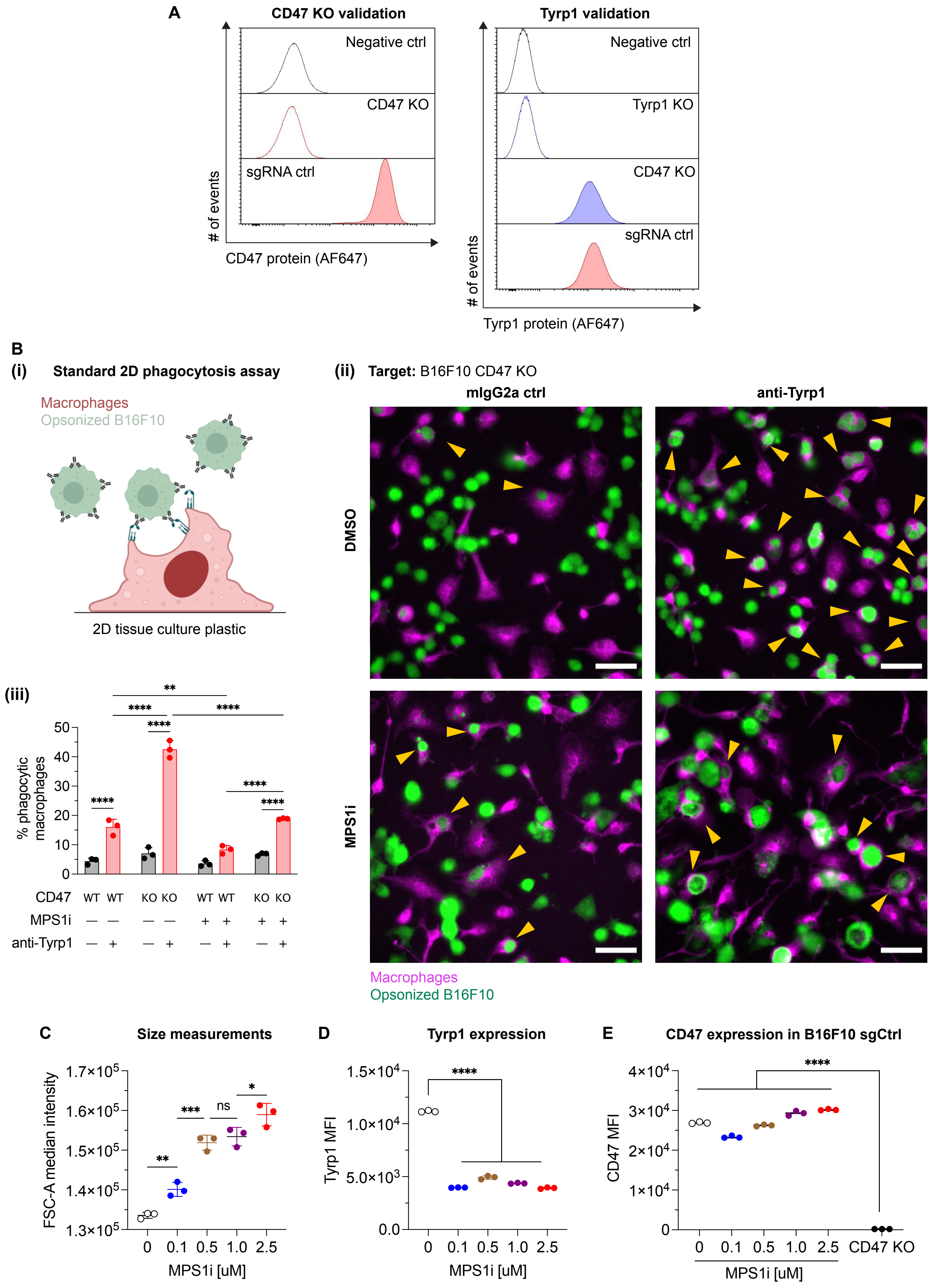

bioRxiv preprint doi: https://doi.org/10.1101/2023.04.02.535275; this version posted April 4, 2023. The copyright holder for this preprint (which was not certified by peer review) is the author/funder, who has granted bioRxiv a license to display the preprint in perpetuity. It is made available under aCC-BY 4.0 International license.

Supplementary Figure 3

MPS1i-induced CIN favors clearance with CD47 ablation and IgG opsonization, regardless of MPS1i concentrtion

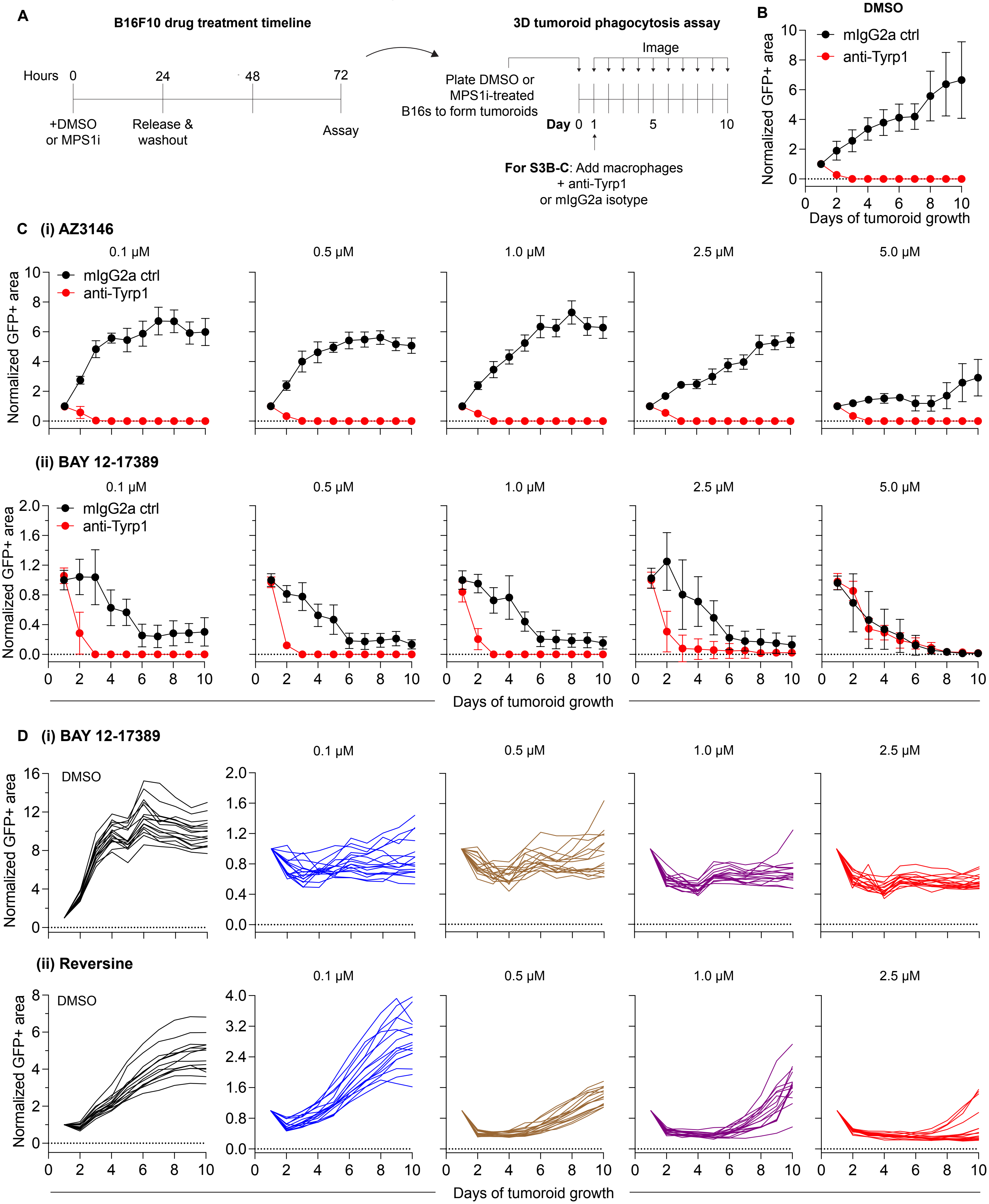

bioRxiv preprint doi: https://doi.org/10.1101/2023.04.02.535275; this version posted April 4, 2023. The copyright holder for this preprint (which was not certified by peer review) is the author/funder, who has granted bioRxiv a license to display the preprint in perpetuity. It is made available under aCC-BY 4.0 International license.

Supplementary Figure 4

MPS1i also induces upregulation of MHC-I class molecules

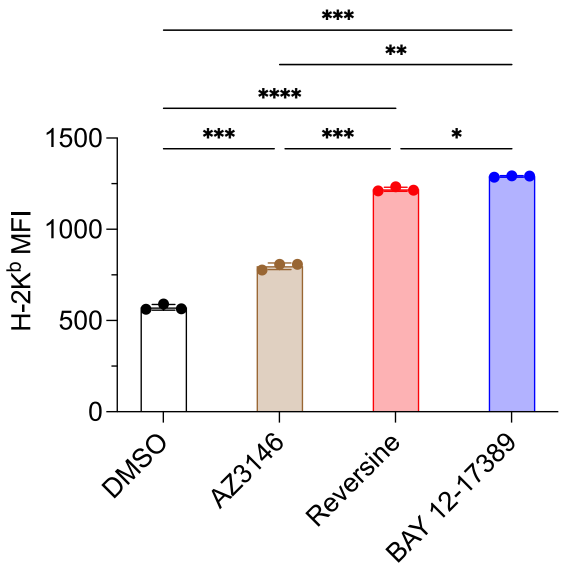

bioRxiv preprint doi: https://doi.org/10.1101/2023.04.02.535275; this version posted April 4, 2023. The copyright holder for this preprint (which was not certified by peer review) is the author/funder, who has granted bioRxiv a license to display the preprint in perpetuity. It is made available under aCC-BY 4.0 International license.

Supplementary Figure 5

Immune infiltrate analysis in MPS1-treated and DMSO-treated tumors

A **Chromosomally stable tumor (DMSO-treated B16F10)**

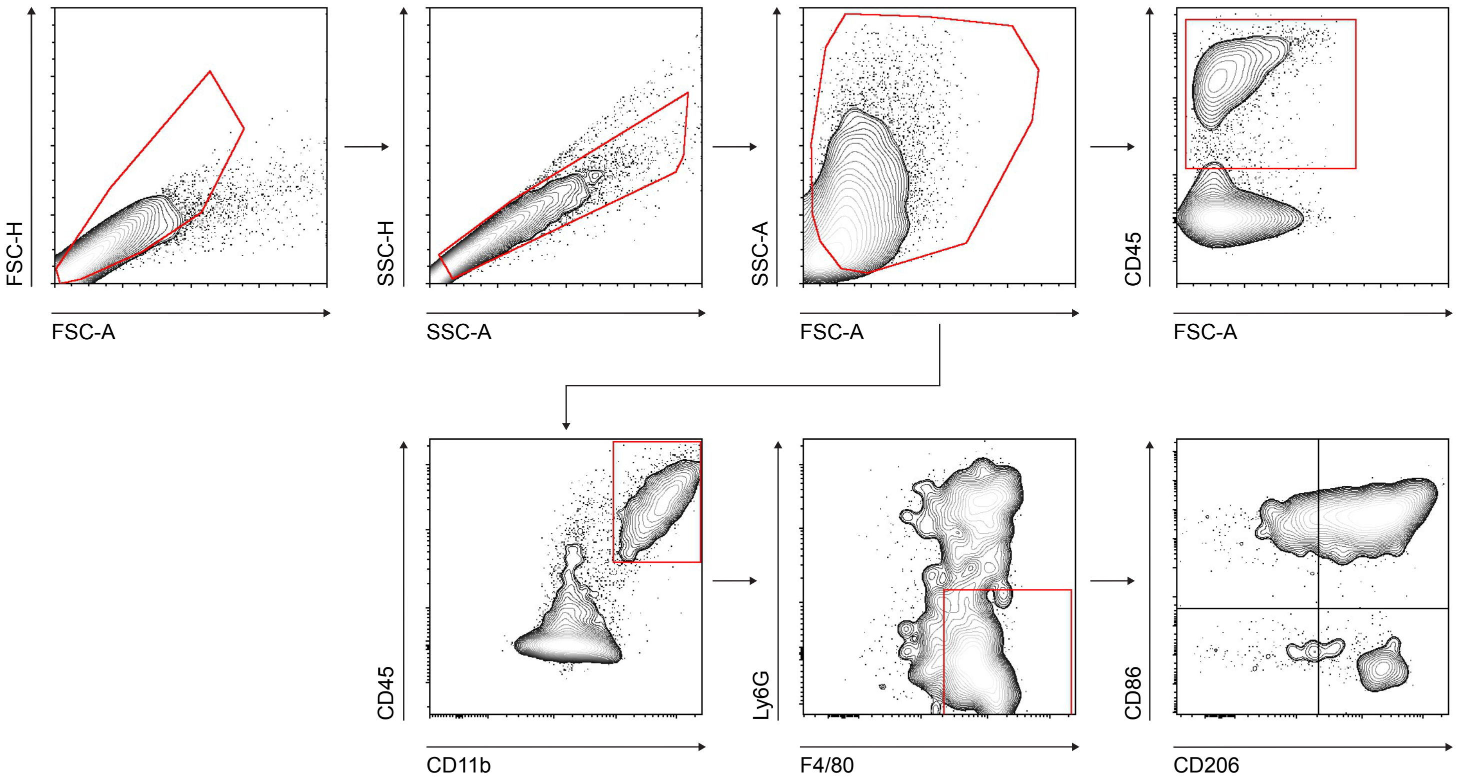

B **Chromosomally unstable tumor (MPS1i-treated B16F10)**

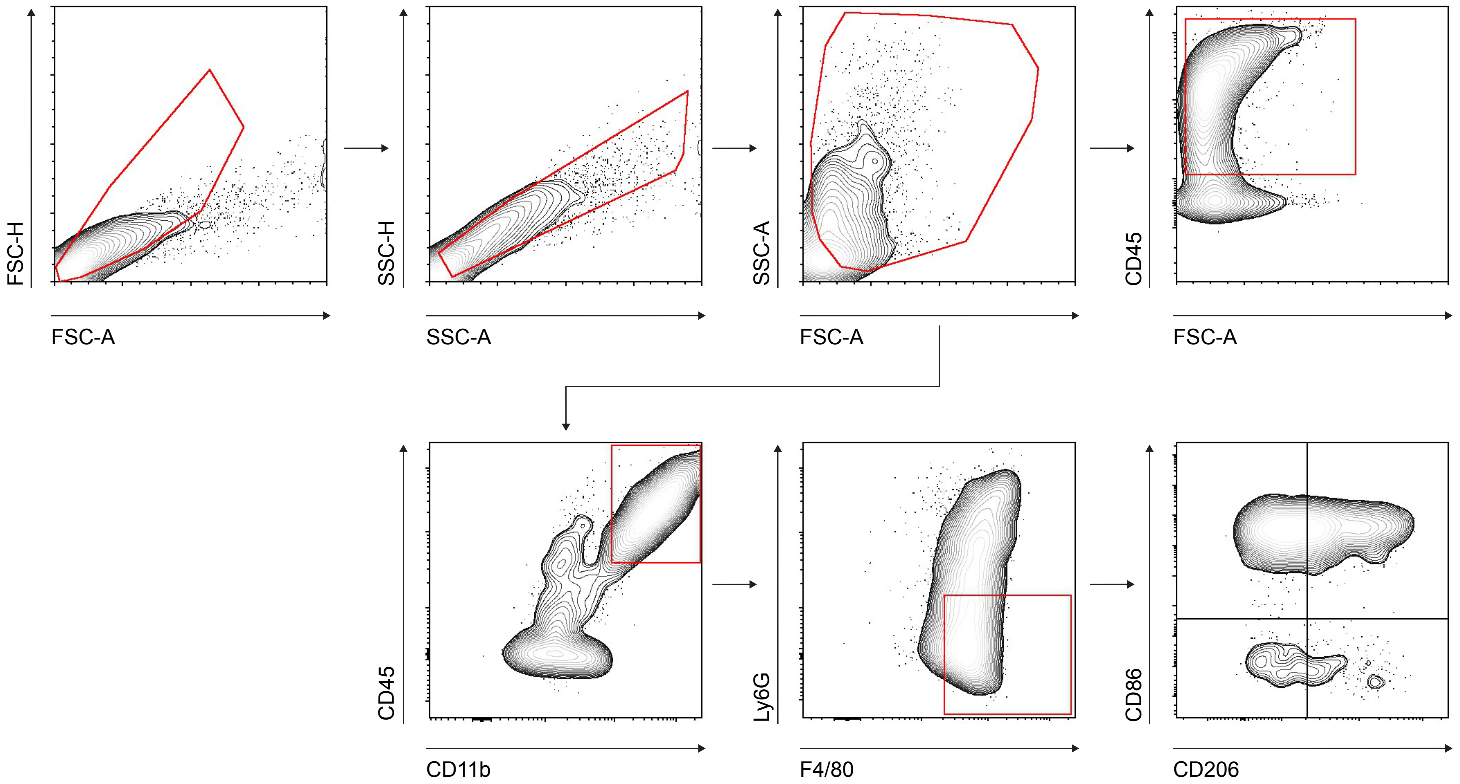

bioRxiv preprint doi: https://doi.org/10.1101/2023.04.02.535275; this version posted April 4, 2023. The copyright holder for this preprint (which was not certified by peer review) is the author/funder, who has granted bioRxiv a license to display the preprint in perpetuity. It is made available under aCC-BY 4.0 International license.

Supplementary Figure 6

MPS1i-induced CIN favors *in vivo* clearance with CD47 ablation and IgG opsonization, regardless of degree of CIN

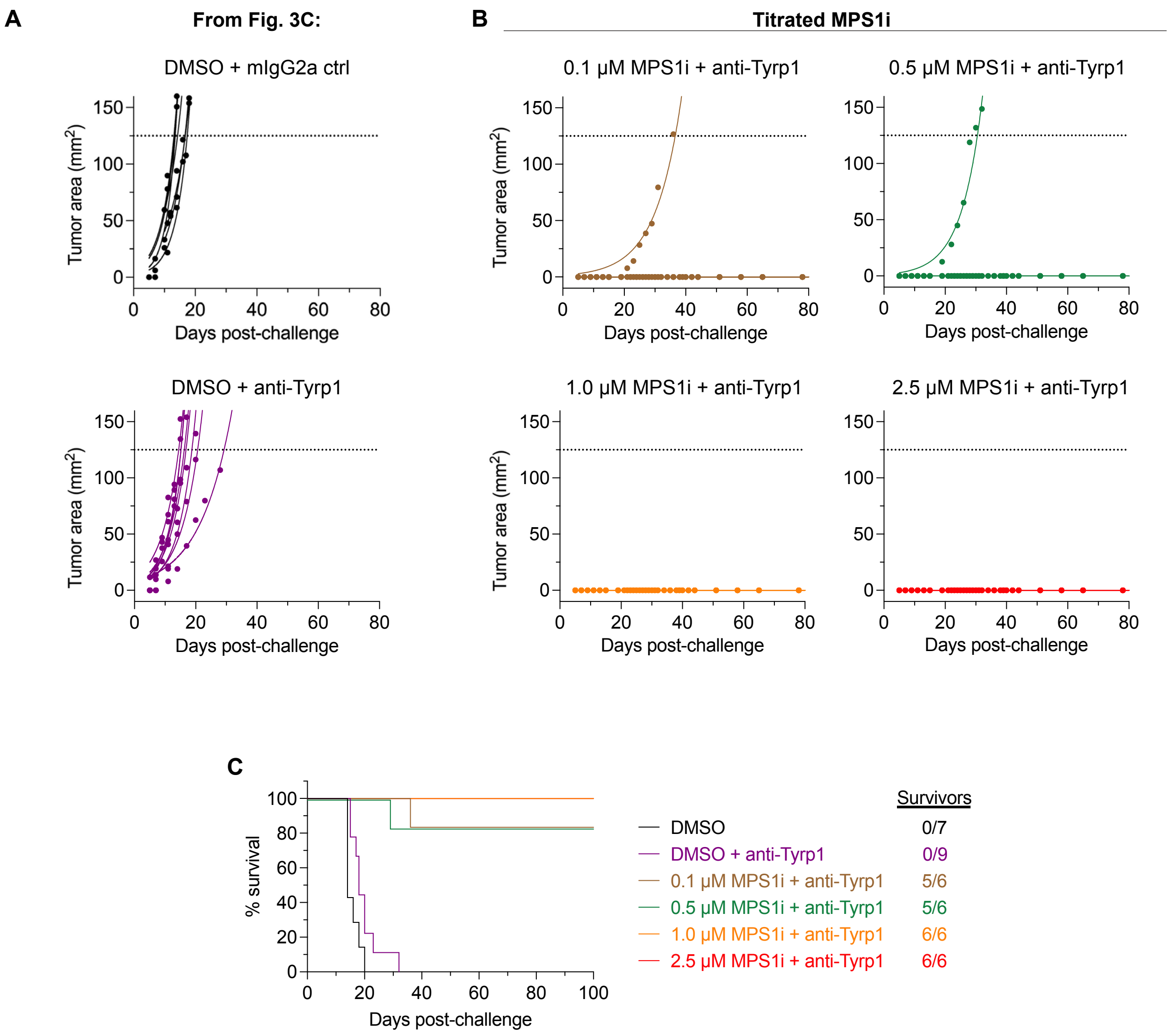

bioRxiv preprint doi: https://doi.org/10.1101/2023.04.02.535275; this version posted April 4, 2023. The copyright holder for this preprint (which was not certified by peer review) is the author/funder, who has granted bioRxiv a license to display the preprint in perpetuity. It is made available under aCC-BY 4.0 International license.

Supplementary Figure 7

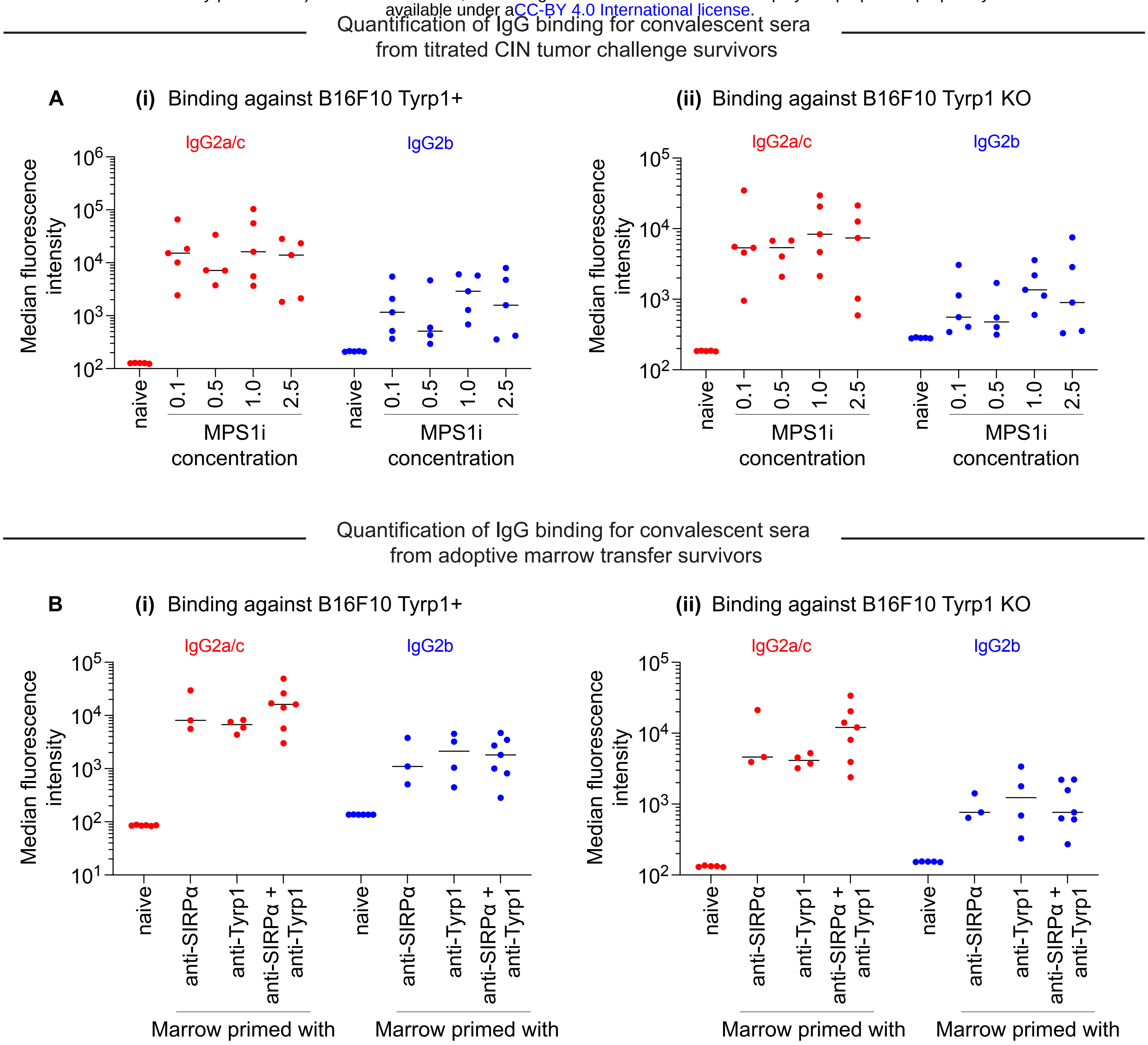

bioRxiv preprint doi: https://doi.org/10.1101/2023.04.02.535275; this version posted April 4, 2023. The copyright holder for this preprint (which was not certified by peer review) is the author/funder, who has granted bioRxiv a license to display the preprint in perpetuity. It is made available under aCC-BY 4.0 International license.

Supplementary Figure 8

Flow cytometry analysis of immune infiltrate in 2nd tumor challenge mice

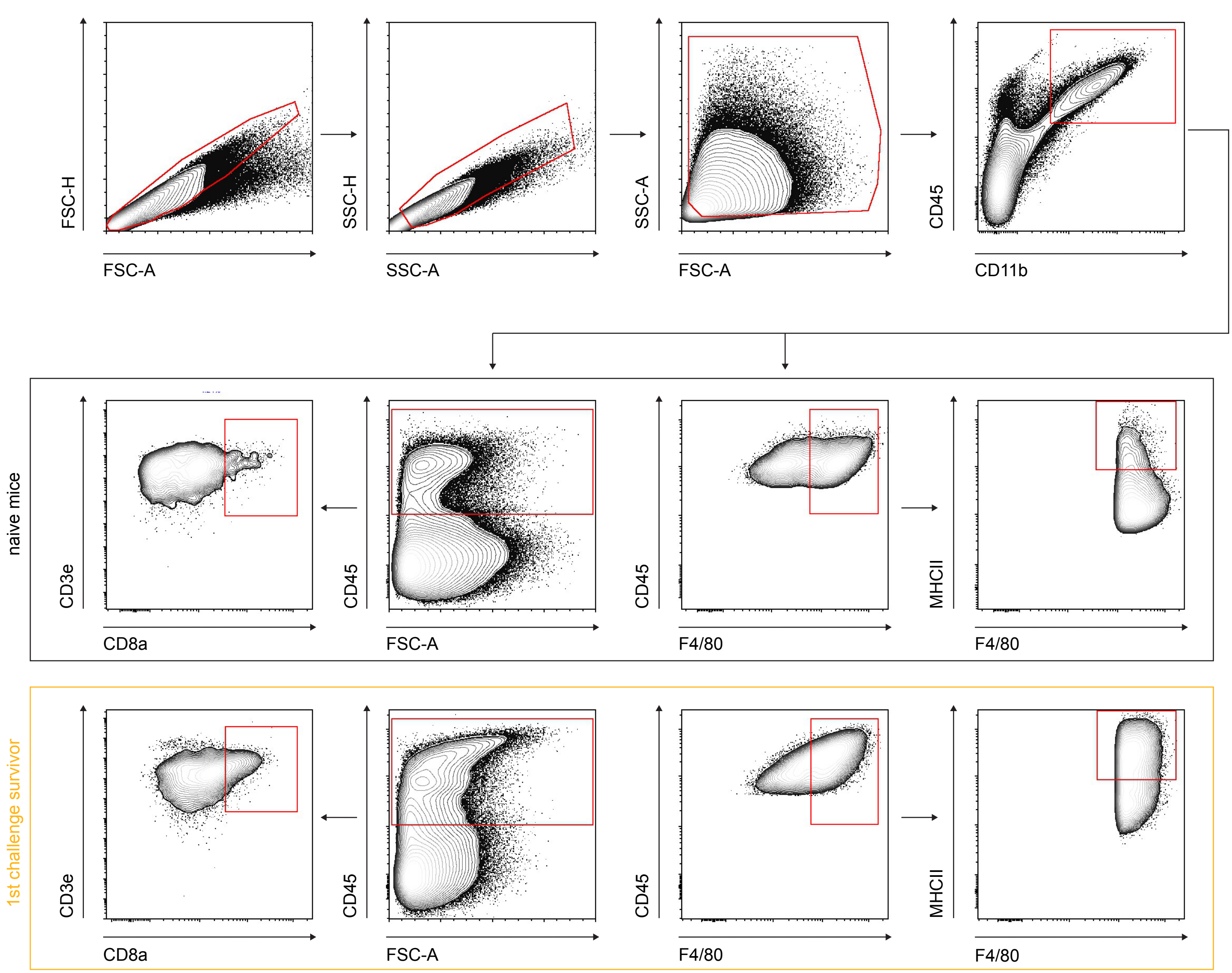

Supplement: Supplement 1 [file NIHPP2023.04.02.535275v1-supplement-1.pdf]
